# Financial Strain Partially Explains Diminished Returns of Parental Education in the ABCD Study

**DOI:** 10.31586/ojn.2024.1129

**Published:** 2024-11-21

**Authors:** Shervin Assari, Hossein Zare

**Affiliations:** 1 Department of Internal Medicine, Charles R. Drew University of Medicine and Science, Los Angeles, CA, United States; 2 Department of Family Medicine, Charles R. Drew University of Medicine and Science, Los Angeles, CA, United States; 3 Department of Urban Public Health, Charles R. Drew University of Medicine and Science, Los Angeles, CA, United States; 4 Marginalization-Related Diminished Returns (MDRs) Center, Los Angeles, CA, United States; 5 Department of Health Policy and Management, Johns Hopkins Bloomberg School of Public Health, Baltimore, MD, United States; 6 School of Business, University of Maryland Global Campus (UMGC), Adelphi, MD, USA

**Keywords:** Parental Education, Cortical Volume, Financial Strain, Racial Disparities, Black Children

## Abstract

**Background:**

Previous research shows that socioeconomic status (SES) positively impacts children’s development, yet the benefits are not equally distributed across racial groups. According to the Minorities’ Diminished Returns (MDRs) framework, Black children tend to experience smaller gains from parental education compared to White children.

**Objective:**

Building on the MDRs framework, this study examines whether high financial strain contributes to the diminished returns of parental education for Black children, using data from the Adolescent Brain Cognitive Development (ABCD) Study. We hypothesized that: (1) there would be a positive effect of parental education on total cortical volume, (2) this effect would be weaker for Black than White children, and (3) higher household financial strain in Black families would mediate the diminished returns of parental education on total cortical volume for Black children.

**Methods:**

Data were drawn from the baseline ABCD Study, focusing on 7,936 9- and 10-year-old children identified as either Black (n = 1,775) or White (n = 6,161). Parental education was the key independent variable, covariates included age, sex, household income, and marital status, race was the moderator, financial strain was the mediator, and total cortical volume was the outcome. Structural Equation Models (SEMs) were employed to examine the associations between parental education and cortical volume, with financial strain as a mediator and race as a moderator.

**Results:**

Higher parental education was associated with greater cortical volume in the pooled sample. However, this effect was significantly weaker for Black children. Financial strain partially mediated the observed diminished returns of parental education.

**Conclusion:**

High financial strain experienced by middle-class Black families partially explains why the association between parental education and child development is weaker in Black than White families. Interventions aimed at enhancing educational quality, increasing employability, expanding access to higher-paying jobs, and reducing labor market discrimination against Black individuals may help address racial inequities in child development in the U.S. Efforts to reduce financial strain should extend beyond low-income populations to also support higher-educated minority families.

## 1. Introduction

Socioeconomic status (SES) is widely recognized as a key factor in shaping children’s developmental outcomes, including brain structure and cognitive functioning [1–5]. Higher parental education, a core component of SES, has been associated with positive outcomes in children, such as increased cortical volume, which supports various cognitive functions. However, research suggests that the developmental and health benefits of parental education are not equally distributed across racial groups. In the United States, the associations between parental education and developmental outcomes tend to be weaker for Black children compared to White children [6–12].

This phenomenon is often referred to as “Minority Diminished Returns” (MDRs) [13], where Black children and other racial and ethnic minorities gain less from parental resources than their White counterparts. These diminished returns have been observed across various outcomes, including educational achievement, physical health, and psychological well-being. While the underlying causes of MDRs are complex, they likely reflect the broader structural inequities faced by Black families, including systemic racism, limited access to high-quality schools, and neighborhood disadvantage, all of which may erode the benefits typically associated with higher SES [14–17].

One potential mechanism underlying these diminished returns is financial strain, which disproportionately affects Black families [18–25], even those with higher levels of parental education. Financial strain, defined as the inability to meet basic financial needs, creates stress that can hinder children’s developmental outcomes. Black families often face higher levels of financial strain due to structural barriers, such as wage gaps, employment discrimination, and limited access to generational wealth. These stressors may diminish the positive effects of parental education on children’s brain development by creating an environment of chronic stress, which is known to negatively impact brain structure and function.

This study aims to test whether financial strain mediates the relationship between parental education and total cortical volume, and whether this mediation differs by race. By exploring the role of financial strain, this research seeks to deepen our understanding of the mechanisms driving racial disparities in brain development and to inform policies aimed at addressing these inequities.

## 2. Methods

### 2.1. Settings and Design

This study utilized data from the Adolescent Brain Cognitive Development (ABCD) Study, a large-scale, longitudinal research initiative designed to examine brain development and child health in the United States. The ABCD study recruited over 11,000 children aged 9-10 years from 21 sites across the country, using a multi-stage probability sampling method to ensure a diverse and representative sample. Data collection involved a combination of neuroimaging, behavioral assessments, and questionnaires completed by both children and their parents. The current analysis leverages cross-sectional baseline data from the ABCD study, focusing on brain structure, socioeconomic factors, and demographic variables.

### 2.2. Sample and Sampling

The initial sample for the ABCD study included 11,878 children. For this analysis, we restricted the sample to children identified by their parents as either Black or White, consistent with our focus on racial disparities. To ensure reliable estimates of brain structure, children with missing or poor-quality neuroimaging data were excluded from the sample. After applying these inclusion criteria, the final analytic sample consisted of 7,936 children (1,775 Black and 6,161 White), which provided sufficient power to detect the hypothesized effects.

### 2.3. Eligibility for the Current Analysis

Eligibility for inclusion in the current analysis required that children meet the following criteria: (1) aged 9-10 years at baseline, (2) identified as Black or White, (3) had data on socioeconomic factors such as parental education and financial strain, and (4) had structural MRI data for total cortical volume. Children with missing key demographic or socioeconomic data were excluded from the analysis.

### 2.4. Measures

#### Predictor (Parental Education):

The key independent variable was parental education, measured as the highest level of education completed by either parent. This was treated as a continuous variable, representing years of completed education.

#### Mediator (Financial Strain):

Financial strain was measured using a parental questionnaire that assessed the family’s ability to meet basic financial needs. Questions included whether the family had difficulty paying bills, affording food, or covering medical expenses. Responses were coded to create a continuous financial strain score, with higher values indicating greater financial difficulties.

#### Covariates (Demographic Factors):

We controlled for several demographic variables that could influence brain development, including the child’s age, sex, household income, and parental marital status. Household income was treated as a continuous variable, while marital status was categorized as married or not married. Age and sex were included to account for normal developmental differences in cortical volume across children.

#### Moderator (Race):

Race was self-identified as was either White (coded as 0) or Black (coded as 1).

#### Outcome (Total Cortical Volume):

The primary outcome of interest was total cortical volume, measured using MRI data collected as part of the ABCD study’s neuroimaging protocol. Cortical volume was calculated by summing the volumes of cortical gray matter across both hemispheres, using FreeSurfer software for brain image processing.

### 2.6. Statistical Analysis

We employed Structural Equation Modeling (SEM) to test the associations between parental education and total cortical volume, with financial strain as a potential mediator and race as a moderator. Three models were specified to evaluate the direct and indirect effects of parental education:

#### Model 1:

We first tested the main effects of parental education on total cortical volume without including any interaction or mediation terms. This model estimated the overall association between parental education and cortical volume across the pooled sample, controlling for the covariates.

#### Model 2:

In the second model, we added an interaction term between race (Black/White) and parental education to test for Minority Diminished Returns (MDRs). This allowed us to assess whether the effect of parental education on cortical volume differed by race, without accounting for the potential mediating effect of financial strain.

#### Model 3:

In the final model, we included both the race-by-parental education interaction and financial strain as a mediator. This model tested whether financial strain partially explained the racial differences in the association between parental education and cortical volume, providing a more comprehensive understanding of the pathways through which SES affects brain development.

All analyses were conducted using SEM in Stata, and we used maximum likelihood estimation to account for missing data. Model fit was assessed using standard indices such as the Comparative Fit Index (CFI) and Root Mean Square Error of Approximation (RMSEA). Significance was evaluated at p < .05.

### 2.7. Ethics

The ABCD study received approval from the Institutional Review Boards (IRBs) at each of the 21 data collection sites. Informed consent was obtained from all parents or legal guardians, and assent was obtained from children before participation. This study’s secondary analysis of de-identified ABCD data was approved by Charles R Drew University of Medicine and Science, ensuring compliance with ethical guidelines for human subjects research [IRB number = 1725413-1].

## 3. Results

Table 1 provides the descriptive statistics for the key study variables. The average age of the children in the sample was 9.48 years (SE = 0.005). In terms of race, 72.9% of the sample identified as White, and 27.1% identified as Black. The sample was fairly balanced by gender, with 47.6% of participants identifying as female and 52.4% as male. Regarding the marital status of the household, 34.0% of children lived in an unwed household, while 66.0% lived in a married household.

Table 2 shows the summary of our three SEMs. In Model 1, parental education had a positive effect on cortical volume (B = 0.085, SE = 0.010, 95% CI = 0.065 to 0.105, p < 0.001), but Black race was negatively associated with total cortical volume (B = −0.223, SE = 0.009, 95% CI = −0.240 to −0.205, p < 0.001). Total family income was also a significant positive predictor (B = 0.113, SE = 0.012, 95% CI = 0.089 to 0.137, p < 0.001). Marital status, however, did not show a significant effect (B = 0.012, SE = 0.010, 95% CI = −0.007 to 0.031, p = 0.203). Age was negatively associated with total cortical volume (B = −0.043, SE = 0.008, 95% CI = −0.058 to −0.028, p < 0.001), while being male was positively associated with total cortical volume (B = 0.418, SE = 0.007, 95% CI = 0.404 to 0.432, p < 0.001)

In Model 2, the interaction between race and parental education was added, showing that the positive association between parental education and cortical volume was significantly weaker for Black children (Education x Black: B = −0.529, SE = 0.149, 95% CI = −0.820 to −0.237, p < 0.001). The main effect of parental education increased slightly (B = 0.100, SE = 0.011, 95% CI = 0.079 to 0.122, p < 0.001). Race (Black) alone had a positive effect in this model (B = 0.312, SE = 0.150, 95% CI = 0.017 to 0.607, p = 0.038).

In Model 3, financial strain was introduced as a mediator. Financial strain was negatively associated with total cortical volume (B = −0.018, SE = 0.009, 95% CI = −0.035 to −0.001, p = 0.038). The interaction between race and parental education remained significant (B = −0.515, SE = 0.149, 95% CI = −0.807 to −0.224, p = 0.001), and the race effect stayed positive (B = 0.300, SE = 0.150, 95% CI = 0.005 to 0.595, p = 0.046). Parental education continued to have a positive effect on cortical volume (B = 0.101, SE = 0.011, 95% CI = 0.080 to 0.123, p < 0.001).

Additionally, total family income (B = −0.355, SE = 0.013, 95% CI = −0.380 to −0.329, p < 0.001), marital status (B = −0.033, SE = 0.010, 95% CI = −0.054 to −0.013, p = 0.001), and parental education (B = −0.039, SE = 0.012, 95% CI = −0.062 to −0.016, p = 0.001) were all negatively associated with financial strain. The interaction between race and parental education was positively associated with financial strain (B = 0.546, SE = 0.161, 95% CI = 0.230 to 0.861, p = 0.001), while Black race alone was negatively associated with financial strain (B = −0.475, SE = 0.163, 95% CI = −0.794 to −0.155, p = 0.004).

## 4. Discussion

This study examined the relationship between parental education and cortical volume in Black and White children, with a particular focus on whether financial strain mediates racial differences in this association. Our findings contribute to a growing body of literature on how socioeconomic resources, such as parental education, impact children’s brain development and how these benefits are unequally distributed across racial groups.

First, we found that parental education was positively associated with total cortical volume across the full sample, confirming previous research on the benefits of higher parental SES for brain development. Children with more educated parents tended to have larger cortical volumes, a finding consistent with evidence that SES-linked resources, such as access to stimulating environments and higher-quality healthcare, contribute to brain growth during critical developmental periods. This main effect highlights the importance of parental education in fostering neurodevelopmental advantages.

However, the results also revealed a significant interaction between race and parental education, providing evidence of Minority Diminished Returns (MDRs) [26–30]. Specifically, while parental education was associated with larger cortical volumes in White children, this association was significantly weaker for Black children. These findings align with previous work suggesting that the benefits of higher SES do not equally extend to Black families. Structural inequalities, including racial discrimination, residential segregation, and unequal access to high-quality schools and healthcare, likely dilute the advantages typically associated with parental education for Black families, contributing to these diminished returns.

The brain is a highly plastic organ, meaning its structure and function can adapt and change in response to environmental influences. This plasticity suggests that the negative effects of financial strain and low SES are potentially reversible. However, reversing these effects requires targeted interventions, such as policy changes, environmental enhancements, and other supportive modalities. Given evidence that low parental education and high financial strain are associated with reduced cortical volume in both White and Black children, there is an urgent need for policies aimed at mitigating these impacts. Interventions should be implemented early, while the brain retains its plasticity, to maximize their effectiveness. Examples of such policies include increasing access to quality education and providing direct financial support to families and communities experiencing economic hardship. These efforts could play a crucial role in promoting healthy brain development and reducing disparities linked to SES.

Further, our analysis identified financial strain as a partial mediator of the diminished returns on parental education for Black children. Black families, even those with higher parental education, were more likely to experience financial strain, which in turn was associated with smaller cortical volumes in their children. Financial strain, which can lead to chronic stress and less investment in educational resources, may offset the expected neurodevelopmental benefits of parental education. This finding suggests that financial difficulties play a key role in explaining the racial disparities in how SES influences brain development.

Financial strain is a major determinant of health disparities in the United States, disproportionately affecting racial and ethnic minorities, particularly Black families [18–25]. Economic hardship has far-reaching effects on physical and mental health, contributing to chronic stress, limited access to healthcare, and poor health outcomes. For families experiencing financial strain, basic needs such as food security, stable housing, and medical care may be compromised, creating conditions that foster the development of health problems. In addition, financial strain often exacerbates stress levels, which can lead to the activation of physiological stress responses, such as increased cortisol levels, that are harmful to the body over time. Chronic exposure to financial stressors has been linked to higher rates of cardiovascular disease, mental health disorders, and other chronic conditions, disproportionately affecting minority communities [18–25]. The persistence of racial wealth gaps, job insecurity, and limited access to economic opportunities means that Black families are more likely to face these financial challenges, which in turn widen existing health disparities. Addressing financial strain through targeted economic policies is crucial for reducing the health inequities that stem from the socioeconomic disadvantages experienced by minority populations in the U.S.

While these findings offer important insights, several limitations should be considered. First, the study is cross-sectional, limiting our ability to draw causal inferences. Longitudinal data would be necessary to better understand how parental education and financial strain over time shape children’s brain development. Second, our measure of financial strain was limited to self-reports, which may not fully capture the broader economic hardships that Black families face. Future research should consider more comprehensive measures of financial hardship, including wealth, debt, and access to social safety nets. Finally, although we focused on parental education, other aspects of SES, such as parental occupation and neighborhood context, could also play a role in shaping racial disparities in cortical volume and should be explored in future studies.

The implications of this study are significant for both policy and practice. Interventions aimed at reducing racial disparities in children’s brain development should not only focus on improving educational attainment for parents but also address the structural barriers that limit the translation of parental education into developmental gains for Black children. Policies designed to reduce financial strain, such as expanding access to financial assistance programs, reducing wage gaps, and addressing employment discrimination, could help mitigate the negative effects of financial stress on children’s brain development. Moreover, investments in early childhood education, particularly in underserved Black communities, may help reduce disparities by providing children with supportive environments that promote brain development.

In conclusion, this study demonstrates that while parental education is generally beneficial for children’s brain development, its effects are not uniform across racial groups. Black children experience diminished returns on parental education, with financial strain partially explaining these disparities. Addressing the structural inequalities that contribute to financial strain and other stressors is essential for ensuring that all children, regardless of race, can fully benefit from the resources their parents provide.

## Figures and Tables

**Figure 1.**
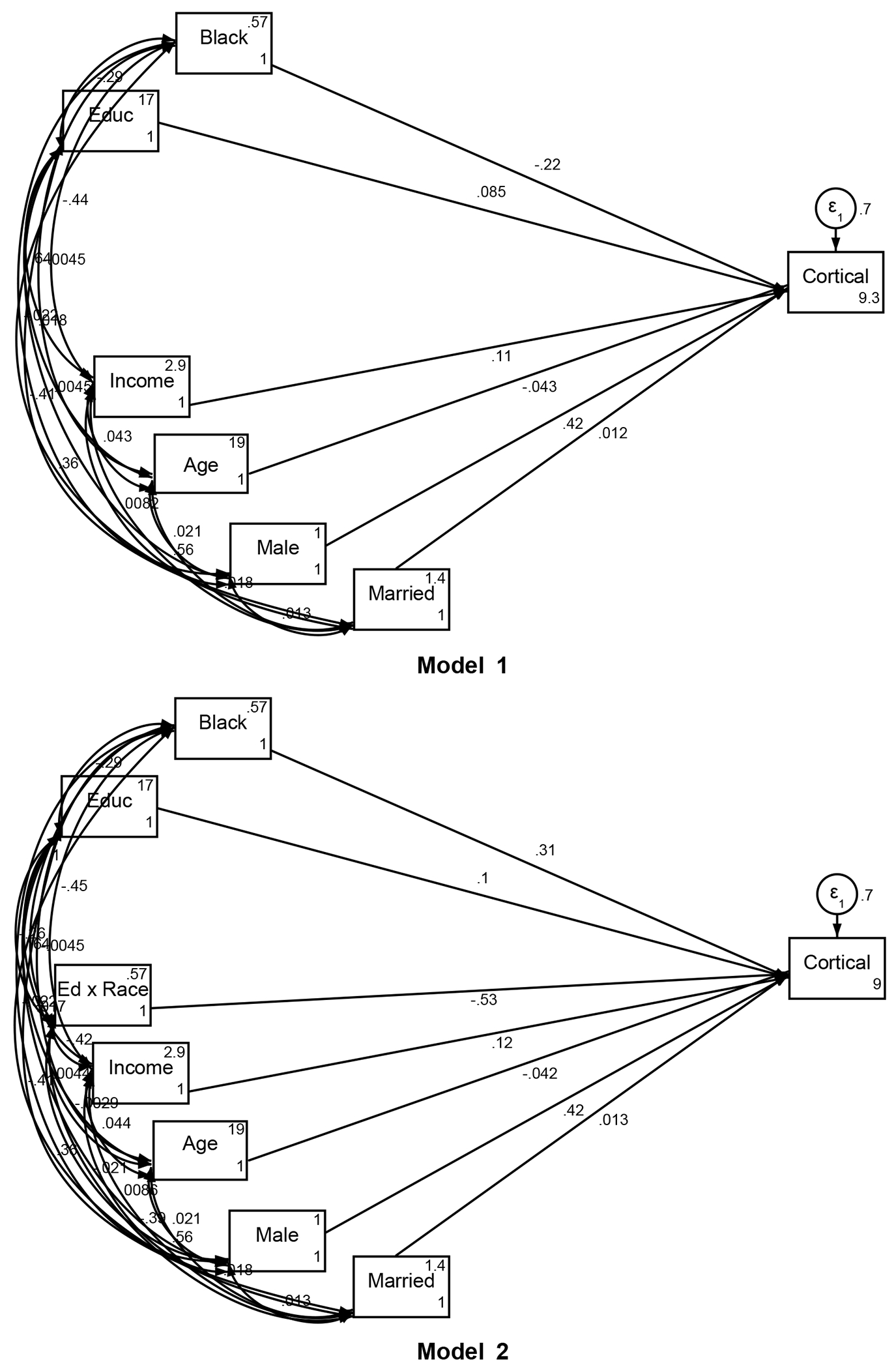

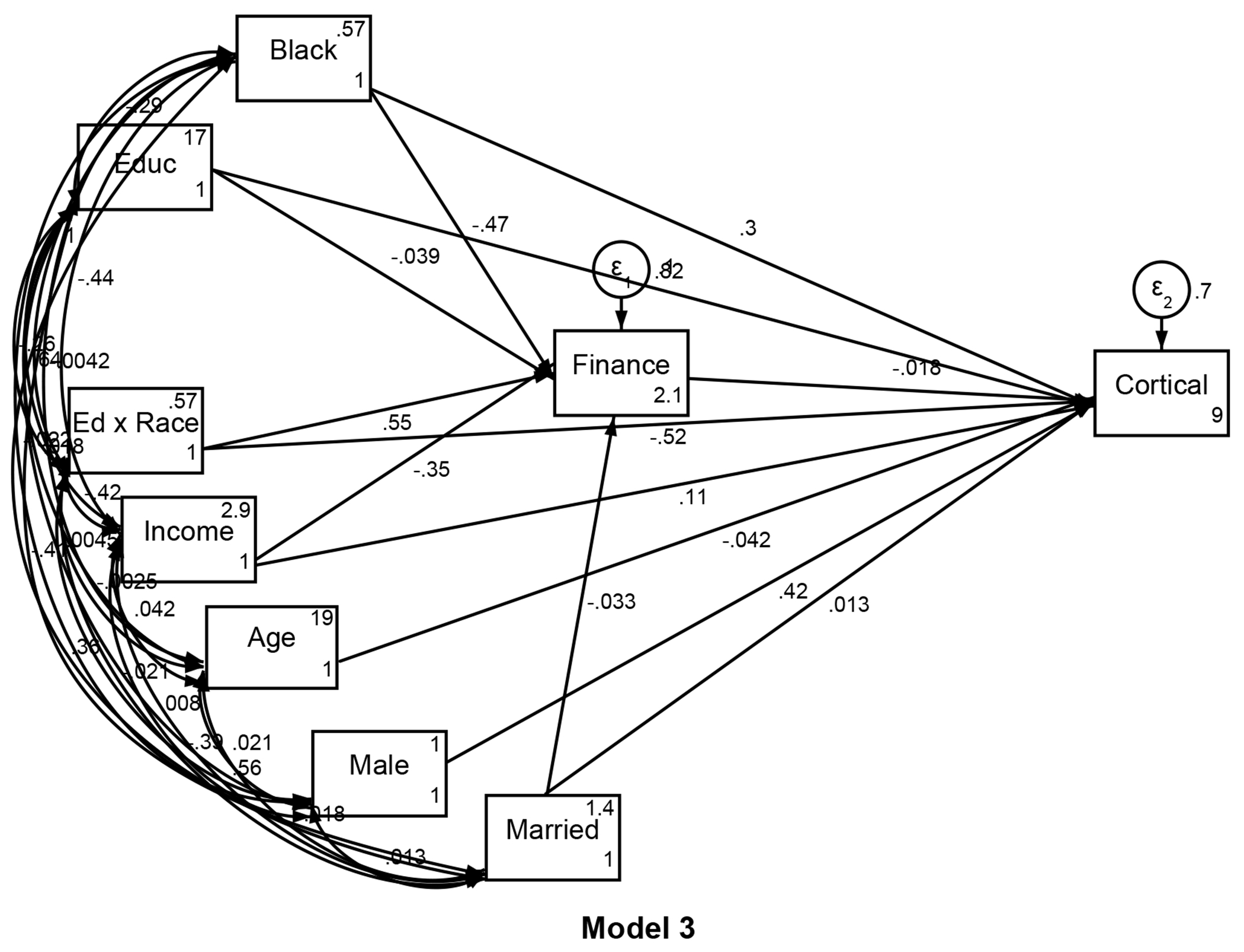
Summary of structural equation models

**Table 1.** Descriptive Data Overall

	Mean	Std. err.	[95% conf.	interval]
				
Age	9.480	0.005	9.470	9.490
	Proportion	SE	[95% conf.	interval]
Race				
White	0.729	0.006	0.717	0.740
Black	0.271	0.006	0.260	0.283
Gender				
Female	0.476	0.007	0.463	0.489
Male	0.524	0.007	0.511	0.537
Marital Status of the Household				
Unwed Household	0.340	0.006	0.327	0.352
Married Household	0.660	0.006	0.648	0.673

**Table 2.** Summary of structural equation models

			B	SE	95%	CI	p
**Model 1**							
Age	→	Total Cortical Volume	−0.043	0.008	−0.058	−0.028	< 0.001
Male	→	Total Cortical Volume	0.418	0.007	0.404	0.432	< 0.001
Total Family Income	→	Total Cortical Volume	0.113	0.012	0.089	0.137	< 0.001
Married Household	→	Total Cortical Volume	0.012	0.010	−0.007	0.031	0.203
Education (Jaeger)	→	Total Cortical Volume	0.085	0.010	0.065	0.105	< 0.001
Race (Black)	→	Total Cortical Volume	−0.223	0.009	−0.240	−0.205	< 0.001
Intercept	→	Total Cortical Volume	9.303	0.226	8.860	9.745	< 0.001
**Model 2**							
Age	→	Total Cortical Volume	−0.042	0.008	−0.058	−0.027	< 0.001
Male	→	Total Cortical Volume	0.418	0.007	0.404	0.432	< 0.001
Total Family Income	→	Total Cortical Volume	0.117	0.012	0.093	0.141	< 0.001
Married Household	→	Total Cortical Volume	0.013	0.010	−0.006	0.032	0.185
Education (Jaeger)	→	Total Cortical Volume	0.100	0.011	0.079	0.122	< 0.001
Education (Jaeger) x Black	→	Total Cortical Volume	−0.529	0.149	−0.820	−0.237	< 0.001
Race (Black)	→	Total Cortical Volume	0.312	0.150	0.017	0.607	0.038
Intercept	→	Total Cortical Volume	9.017	0.239	8.548	9.486	< 0.001
**Model 3**							
Financial Strain (Mean)	→	Total Cortical Volume	−0.018	0.009	−0.035	−0.001	0.038
Age	→	Total Cortical Volume	−0.042	0.008	−0.057	−0.027	< 0.001
Male	→	Total Cortical Volume	0.418	0.007	0.404	0.432	< 0.001
Total Family Income	→	Total Cortical Volume	0.109	0.013	0.084	0.134	< 0.001
Married Household	→	Total Cortical Volume	0.013	0.010	−0.006	0.032	0.182
Education (Jaeger)	→	Total Cortical Volume	0.101	0.011	0.080	0.123	< 0.001
Education (Jaeger) x Black	→	Total Cortical Volume	−0.515	0.149	−0.807	−0.224	0.001
Race (Black)	→	Total Cortical Volume	0.300	0.150	0.005	0.595	0.046
Intercept	→	Total Cortical Volume	9.024	0.239	8.555	9.493	< 0.001
							
Total Family Income	→	Financial Strain	−0.355	0.013	−0.380	−0.329	< 0.001
Married Household	→	Financial Strain	−0.033	0.010	−0.054	−0.013	0.001
Education (Jaeger)	→	Financial Strain	−0.039	0.012	−0.062	−0.016	0.001
Education (Jaeger) x Black	→	Financial Strain	0.546	0.161	0.230	0.861	0.001
Race (Black)	→	Financial Strain	−0.475	0.163	−0.794	−0.155	0.004
Intercept	→	Financial Strain	2.124	0.188	1.756	2.493	< 0.001
